# Reactive Transport Modeling of Subaqueous Sediment Caps and Implications for the Long-Term Fate of Arsenic, Mercury, and Methylmercury

**DOI:** 10.1007/s10498-012-9165-4

**Published:** 2012-04-27

**Authors:** Brad A. Bessinger, Dimitri Vlassopoulos, Susana Serrano, Peggy A. O’Day

**Affiliations:** 1 S.S. Papadopulos and Associates, 19215 SE 34th St., Suite 106-370, Camas, WA 98607 USA; 2 Anchor QEA, 421 SW Sixth Avenue, Suite 750, Portland, OR 97204 USA; 3 School of Natural Sciences, University of California, 5200 North Lake Rd, Merced, CA 95343 USA; 4 Present Address: Institute for Agricultural Sciences, Consejo Superior de Investigaciones Científicas (CSIC), Serrano 115-dup, 28006 Madrid, Spain

**Keywords:** Sediment cap, Remediation, Reactive transport, Biogeochemical kinetics

## Abstract

A 1-D biogeochemical reactive transport model with a full set of equilibrium and kinetic biogeochemical reactions was developed to simulate the fate and transport of arsenic and mercury in subaqueous sediment caps. Model simulations (50 years) were performed for freshwater and estuarine scenarios with an anaerobic porewater and either a diffusion-only or a diffusion plus 0.1-m/year upward advective flux through the cap. A biological habitat layer in the top 0.15 m of the cap was simulated with the addition of organic carbon. For arsenic, the generation of sulfate-reducing conditions limits the formation of iron oxide phases available for adsorption. As a result, subaqueous sediment caps may be relatively ineffective for mitigating contaminant arsenic migration when influent concentrations are high and sorption capacity is insufficient. For mercury, sulfate reduction promotes the precipitation of metacinnabar (HgS) below the habitat layer, and associated fluxes across the sediment–water interface are low. As such, cap thickness is a key design parameter that can be adjusted to control the depth below the sediment–water interface at which mercury sulfide precipitates. The highest dissolved methylmercury concentrations occur in the habitat layer in estuarine environments under conditions of advecting porewater, but the highest sediment concentrations are predicted to occur in freshwater environments due to sorption on sediment organic matter. Site-specific reactive transport simulations are a powerful tool for identifying the major controls on sediment- and porewater-contaminant arsenic and mercury concentrations that result from coupling between physical conditions and biologically mediated chemical reactions.

## Introduction

Subaqueous sand caps are a remedial alternative for managing contaminated sediments that have received considerable attention in recent years (Azcue et al. 1998; Palermo 1998; Wang et al. 2004; USEPA 2005). This technology involves the placement of clean sediment or sand over contaminated material to physically isolate chemical constituents from ecological receptors. Although relatively simple and optimum in low-energy environmental conditions (e.g., lakes, estuaries, low-velocity river reaches), the effectiveness of capping can be compromised when there are diffusive and/or advective fluxes of chemical contaminants from underlying groundwater (Liu et al. 2001). Depending on the magnitude of these fluxes, successful mitigation may require (1) addition of mineral and organic substrates for adsorption (Moo-Young et al. 2001; Ying and Axe 2005; Viana et al. 2008), (2) favorable geochemical conditions that promote precipitation (or co-precipitation) of metals as crystalline and/or amorphous compounds (Sengor et al. 2007), and/or (3) addition of reactive chemical amendments to facilitate sequestration by adsorption or dissolution/precipitation (Jacobs and Forstner 1999; Yang et al. 2007; Kumpiene et al. 2008).

To simulate the effectiveness of a sediment cap for mitigating contaminants, it is important to consider porewater geochemistry and cap mineralogy because these parameters ultimately govern the partitioning of metals between the aqueous and solid phases. Studies indicate that porewater and mineral phases may evolve over time in capped sediments due to the establishment of microbial and macrofaunal populations. Redox stratification associated with biological activity can result in reductive dissolution of iron oxides, the precipitation of metal sulfides, and/or the formation of methylmercury (Slowey and Brown 2007; Himmelheber et al. 2008; Johnson et al. 2010).

Within the context of simulating sediment cap performance, geochemical and reactive transport modeling is a useful tool because it allows laboratory results to be extrapolated to long timescales. Although a number of models have been developed to understand contaminant transport and fate in subaqueous caps (Alshawabkeh et al. 2005; Lampert and Reible 2009; Viana et al. 2008; Arega and Hayter 2008; Go et al. 2009), most have focused on describing physical processes such as consolidation, bioturbation, advection, and dispersion. Geochemical and biogeochemical processes have generally been simplified in these models and have often relied on assumed distribution coefficients, rather than examining a complete set of integrated equilibrium and kinetic chemical reactions, to represent partitioning and fate.

The objective of the present study was to use a biogeochemical reactive transport model to simulate the mineralogical evolution and long-term fate of redox-active contaminants in a subaqueous sediment cap. Arsenic and mercury were selected for analysis because they are common contaminants in sediment and possess dissimilar geochemistry. Arsenic predominantly occurs as an oxyanion aqueous species, either arsenite or arsenate, and its partitioning to sediment is affected by the presence of iron oxide and arsenic sulfide phases. An important limitation on the effectiveness of capping of arsenic-contaminated sediment is its potentially high solubility under anoxic conditions (Mucci et al. 2000; O’Day et al. 2004). Mercury, in contrast, is a siderophile element that most commonly occurs in groundwaters and porewaters as dissolved inorganic or organic sulfide complexes. Although mercury can be sequestered in relatively insoluble sulfide phases [HgS(s) as cinnabar or metacinnabar], mercury can also be stabilized in solution by complexation with natural organic ligands and sulfide (Skyllberg 2008; Slowey and Brown 2007; Slowey 2010). Mercury may become methylated by sulfate- (and/or iron-) reducing bacteria that populate sediment caps (Ullrich et al. 2001), which is a key step in the production of bioavailable methyl mercury species. This work is preceded by similar studies that have simulated geochemical processes near the sediment–water interface (Dueri et al. 2003; Canavan et al. 2007; Jung et al. 2009; Couture et al. 2010) but did not specifically evaluate contaminant transport in sediment caps associated with contaminant remediation. A comprehensive review and compilation of thermodynamic and kinetic data were performed to construct the database for the reactive transport model presented here. Simulations of the 1-D transport of arsenic- and mercury-contaminated porewater into a clean (quartz) sand cap undergoing biogeochemical reduction near the sediment–water interface were used to (1) assess the relative effectiveness of an unamended cap for immobilizing metals and thus protect ecological receptors in the overlying aqueous environment and (2) identify key factors that should be considered during cap design.

## Methods

### Model Description

1-D reactive transport simulations were performed using the USGS-supported geochemical software PHREEQC (Parkhurst and Appelo 1999). Chemical processes included in the model were heterogeneous and homogeneous equilibrium speciation reactions, kinetic-based reactions describing biodegradation of organic carbon, reduction–oxidation (redox) transformations, mercury methylation, and demethylation. Physical processes included porewater diffusion, with and without porewater advective flow.

**Fig. 1:**
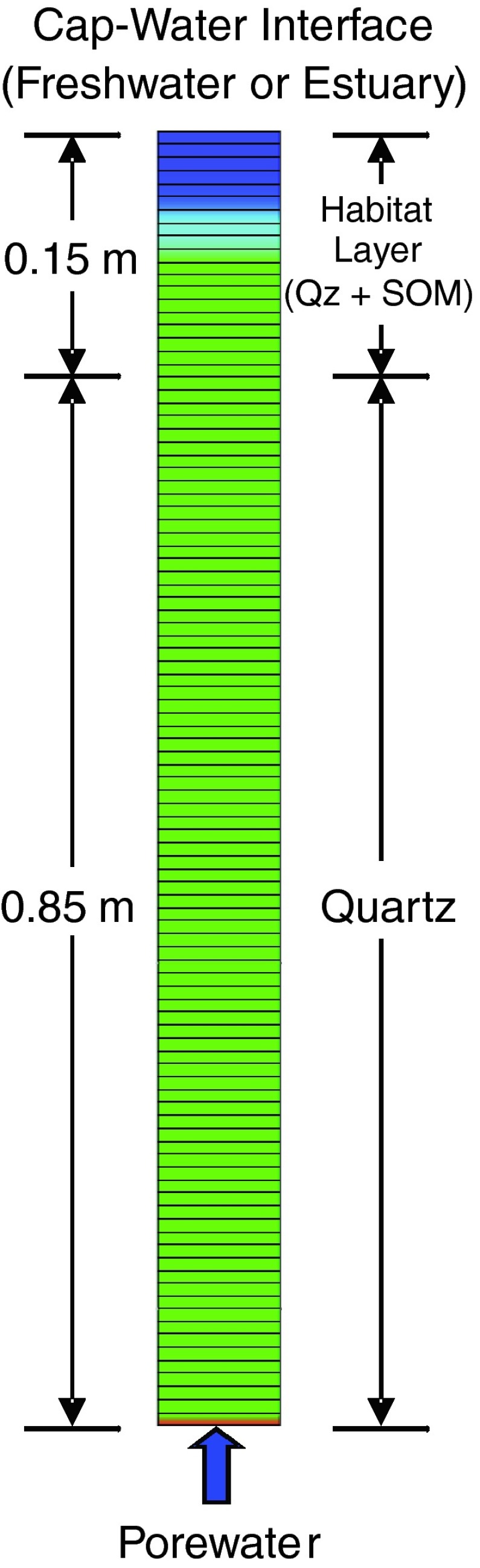
PHREEQC model domain showing grid discretization, *upper* and *lower* boundary conditions, and sediment cap composition

The model simulated a 1-meter-thick quartz sand cap emplaced on top of a layer of contaminated sediment in either an estuarine (salt water) or freshwater setting. The model domain was discretized into 100 grid cells, and constant concentration boundary conditions were applied at the ends (Fig. 1). The lower boundary consisted of an anaerobic porewater with elevated arsenic (10^−3.9^ M, or 10 mg/L) and mercury (10^−7.3^ M, or 10 µg/L), and the upper boundary and initial porewater composition consisted of either salt water or freshwater with no contamination (Table 1). The initial conditions within the cap consisted of entrained water with a chemical composition identical to oxidized surface water (either salt or fresh). The cap was also assumed to include a maximum of 1 % sediment organic matter (SOM) at the sediment–water interface, with the SOM concentration decreasing exponentially with depth in the top 0.15 m of the cap to simulate a habitat layer (Canavan et al. 2006). Porewater was allowed to diffuse and/or advect through the cap for a period of 50 years (Table 2). In some model simulations, the effect of background iron oxide concentrations on contaminant transport and fate was investigated by assuming an initial coating of goethite on the quartz sand in the cap (0.01 mol goethite/kg_sediment_) (Table 2). Model simulations were also performed using influent porewater with a low concentration of arsenic (10^−5.2^ M, or 0.5 mg/L) and mercury (10^−8.6^ M, or 0.5 µg/L) to examine model response to input concentration (Table 3 summarizes the five different estuarine and freshwater model scenarios).

**Table 1:** Chemical composition of aqueous solutions used in cap model

Parameter	Estuary	Freshwater	Groundwater	Units	Comment
pH	8.0	7.0	7.0	Log activity (H^+^)	
Pe	12.6	13.6	−2.5	Log activity (e^−^)	a
As	0.0	0.0	−5.2 or −3.9	Log M	b
C	−3.1	−4.2	−4.5	Log M	
Ca	−2.0	−3.6	−3.6	Log M	
Cl	−1.0	−3.0	−3.0	Log M	a
CO_2_ (g)	−3.5	−3.5	−3.5	Log SI	c
O_2_ (g)	−0.75	−0.75	–	Log SI	c
DOM	−4.7	−4.7	−3.7	Log M	d
Fe	−6.3	−6.3	−5.6	Log M	e
Hg	0.0	0.0	−8.6 or −7.3	Log M	f
N	−5.0	−6.0	−8.0	Log M	
Na	−1.0	−3.3	−3.3	Log M	
S	−2.0	−5.0	−5.0	Log M	g
Si	−4.0	−4.0	−4.0	Log M	h

^a^Groundwater at iron-reducing conditions is charge balanced by chloride

^b^Initial As(III)/As(V) speciation based on thermodynamic equilibrium; range is 0.5–10 mg/L

^c^Set by saturation index (SI)

^d^DOM equivalent to a dissolved organic carbon concentration of 1–10 mg/L with concentrations of thiol sites of 4.7 × 10^−8^–4.7 × 10^−7^ M

^e^Fe(II)/Fe(III) includes a goethite buffering phase for surface water (undersaturated for groundwater)

^f^Reported as total Hg(II) concentration with no methylmercury initially present; range is 0.5–10 µg/L

^g^S(−II)/S(VI) speciation based on thermodynamic equilibrium; no elemental sulfur included in the model

^h^Solution assumed to be in equilibrium with quartz

**Table 2:** Physical and chemical description of quartz sand cap

Parameter	Value	Units	Comment
Chemical composition			
Quartz	11.1	mol/kg	
Fe(III)-oxide	0 or 0.01	mol/kg	a
SOM	0–0.3	mol/kg	b
Flow model parameters			
Column height	1	m	
Porosity	0.4	–	
Hydraulic conductivity	1 × 10^−6^	m/s	
Diffusion coefficient	3 × 10^−10^	m^2^/s	c
Longitudinal dispersivity	0	m	
Groundwater flowrate	0–0.1	m/year	

^a^Simulations performed with no Fe(III)-oxide, or with Fe(III)-oxide modeled as goethite coatings on quartz at 0.01 mol_goethite_/kg_quartz_ based on Kent and Fox (2004), Knapp et al. (2002)

^b^Maximum SOM value occurs at the sediment–water interface with concentrations reduced exponentially as a function of depth using bioturbation equations of Canavan et al. (2006) and an assumed maximum depth of OM of 0.15 m

^c^Composite diffusion coefficient for all aqueous species (Appelo and Postma 2005)

**Table 3:** Description of model scenarios

Model scenario	As_inp_	Hg_inp_	Comments
	Log M	Log M	
Estuarine			
E1: diffusion, high concentration	−3.9	−7.3	Base scenario
E2: diffusion + advection	−3.9	−7.3	Groundwater advection at a rate of 0.1 m/year
E3: diffusion + goethite	−3.9	−7.3	Goethite coating on quartz in cap (0.01 mol_goethite_/kg)^a^
E4: diffusion, low concentration	−5.2	−8.6	Lower groundwater contaminant concentrations
Freshwater			
F1: diffusion, high concentration	−3.9	−7.3	Base scenario
F2: diffusion + advection	−3.9	−7.3	Groundwater advection at a rate of 0.1 m/year
F3: diffusion + goethite	−3.9	−7.3	Goethite coating on quartz in cap (0.01 mol_goethite_/kg)^a^
F4: diffusion, low concentration	−5.2	−8.6	Lower groundwater contaminant concentrations

^a^Kent and Fox (2004), Knapp et al. (2002)

### Thermodynamic Database

Equilibrium speciation calculations in PHREEQC utilized the default LLNL thermodynamic database (Delany and Lundeen 1990) with the following modifications. (1) Addition of an internally consistent thermodynamic database for arsenic aqueous species and minerals (Nordstrom and Archer 2003; Vlassopoulos et al. 2010), (2) inclusion of recently compiled thermodynamic values for inorganic (Powell et al. 2005) and organic mercury aqueous species (Skyllberg 2008), and (3) inclusion of equilibrium constants for FeS(s) and FeS(aq) (Rickard and Luther 2007).

#### Arsenic

Equilibrium constants for arsenic used in this study are reported in “Appendix 1.” Values for arsenic hydroxide and oxyanion species were taken from Nordstrom and Archer (2003), after adjusting the reported values for consistency with the LLNL database (Delany and Lundeen 1990). The sulfide speciation scheme and corresponding equilibrium constants of Vlassopoulos et al. (2010) were used for dissolved arsenic sulfide complexes. Adsorption of dissolved arsenate and arsenite to iron oxide phases was modeled using surface complexation constants and default surface site concentrations reported in the LLNL database for ferrihydrite from Dzombak and Morel (1990). Because model results indicated that goethite and magnetite were thermodynamically stable rather than ferrihydrite, the concentration of surface sites was reduced by a factor of 10 to account for differences in reactive surface area of the crystalline iron oxides (Dixit and Hering 2003). No adsorption to iron sulfide minerals was included in the model because prior studies indicated that precipitation of a realgar-type mineral phase is the dominant mode of arsenic attenuation in the presence of mackinawite (Gallegos et al. 2007) and because of a lack of surface complexation constants for sorption on sulfides consistent with the database. Realgar and orpiment were allowed to precipitate at thermodynamic equilibrium using the solubility constants reported in Vlassopoulos et al. (2010).

#### Mercury

Equilibrium constants for reactions with mercury are reported in “Appendix 2.” Prior studies have discussed the uncertainty in the value of the equilibrium constant for HgS(aq) (written as the species HOHgSH^0^ in “Appendix 2”). As discussed in Skyllberg (2008), experimental conditions designed to measure the formation constant for this species likely included colloidal mercury, which results in an overestimation of the equilibrium constant for dissolved mercury sulfide. Although the equilibrium formation constant for this species has also been estimated theoretically (Dyrssen and Wedborg 1986), this value has been shown to underestimate methylmercury concentrations in the presence of HgS(s) (Drott et al. 2007). Model results of this study predicted that metacinnabar was a stable phase. Therefore, the experimentally derived value of Skyllberg (2008) was used for HOHgSH^0^ in order to obtain a more accurate representation of methylmercury concentrations.

Sorption of mercury and methylmercury on SOM was included in the model by modifying equilibrium reactions for the formation of dissolved mercury-organic complexes (“Appendix 2”). This modification was accomplished by converting the aqueous complexation reactions reported in Skyllberg (2008) to exchange constants that conform to the Gaines-Thomas convention (Appelo and Postma 2005). This conversion depends on the concentration of total thiol (–SH) surface sites on SOM and, to a lesser extent, the protonated fraction of these sites. The reported values in “Appendix 2” are conditional stability constants assuming a concentration of thiol sites associated with SOM of 1.9 × 10^−3^ mol/L water. The abundance of thiol-type functional sites associated with organic matter was set to 0.047 meq/g organic matter (meq/g_OM_) for both dissolved organic matter (DOM) and solid-phase SOM (Skyllberg 2008).

Mercury polysulfide complexes were not included in the model because (1) elemental sulfur was not predicted to form in the cap and (2) formation of these complexes is very weak compared with HgS(aq) in the presence of metacinnabar (Benoit et al. 1999; Jay et al. 2000; Drott et al. 2007). Mercury adsorption to iron sulfide minerals was excluded because the predominant mode of mercury attenuation in the presence of mackinawite is the precipitation of a HgS mineral (in this case, metacinnabar) rather than sorption (Skyllberg and Drott 2010).

### Biogeochemical Reactions and Rates

Biodegradation of two organic carbon fractions was included in the model: (1) DOM, originating in both surface water and porewater, and (2) SOM, originating from surface water and mixed within the top 0.15 m of the sediment cap by bioturbating organisms (Boudreau 1998; Canavan et al. 2006). Concentrations are given in Tables 1 and 2, respectively.

The Monod-type description of biogeochemical reactions and rates in the model, including both primary reduction and secondary oxidation reactions (Table 4), was based on prior studies (Van Cappellen and Wang 1996; Hunter et al. 1998; Canavan et al. 2006). The overall degradation rate of organic matter (OM), including either SOM or DOM, is the sum of individual reaction rates [*R*_*i*_] of successive terminal electron acceptors (EA_*i*_):

1 $$ \begin{gathered} R_{i} = k_{\text{OM}} \left( {1 - \sum\limits_{0}^{i - 1} {f_{i} } } \right)\frac{{\left[ {{\text{EA}}_{i} } \right]}}{x}\quad {\text{and}} \hfill \\ \left\{ {\begin{array}{*{20}c} {x = 1} & {{\text{for}}\quad \left[ {{\text{EA}}_{i} } \right] > K_{{{\text{EA}},i}} } \\ {x = K_{\text{EA}} } & {{\text{for}}\quad \left[ {{\text{EA}}_{i} } \right] < K_{{{\text{EA}},i}} } \\ \end{array} } \right\} \hfill \\ \end{gathered} $$

where *k*_OM_ is the rate constant for OM degradation, *f*_*i*_ is the fraction of electrons consumed by the *i*th primary reduction half-reaction, [EA_*i*_] is the concentration of the *i*th terminal electron acceptor species [EA], and *K*_EA,*i*_ is the limiting concentration for the respective electron acceptor. As shown in Eq. 1, when the concentration of [EA] is below its limiting value *K*_EA_, the corresponding primary reduction rate is reduced. Rate constants (*k*_*i*_) and limiting concentrations (*K*_EA_) used in the model are reported in Table 5. The biodegradation rate constants for SOM and DOM were set to 0.002 and 0.0001 year^−1^, respectively, based on literature review (Canfield et al. 1993; Hulthe et al. 1998; Fossing et al. 2004; Arzayus and Canuel 2005; Wallmann et al. 2006; Komada et al. 2004). Although the biogeochemical rate constants used in this study were not explicitly calibrated, all model parameters were selected to be within the range of values observed at field sites in order to be generally applicable.

**Table 4:** Kinetic reactions used in model simulations

Description	Reaction	Rate formulation [*R*_*i*_]	Comment
Primary redox reactions			
1. Aerobic respiration	CH_2_O(NH_3_)_0.15_ + O_2_ + 0.15 H^+^ → CO_2_ + H_2_O + 0.15 NH_4_^+^	*f*_O2_ × *k*_OM_ × [OM] × accel	a,b
2. Denitrification	CH_2_O(NH_3_)_0.15_ + 0.8 NO_3_^−^ → 0.4 N_2_ + 0.05 CO_2_ + 0.95 HCO_3_^−^ + 0.45 H_2_O + 0.15 NH_4_^+^	*f*_NO3_ × *k*_OM_ × [OM] × accel	a,b
3. Arsenate reduction	CH_2_O(NH_3_)_0.15_ + 2 HAsO_4_^2−^ + 4.15 H^+^ → 2 H_3_AsO_3_ + CO_2_ + H_2_O + 0.15 NH_4_^+^	*f*_As(V)_ × *k*_OM_ × [OM]	a
4. Fe-oxide reduction	CH_2_O(NH_3_)_0.15_ + 4 FeOOH + 8.15 H^+^ → 4 Fe^2+^ + CO_2_ + 7 H_2_O + 0.15 NH_4_^+^	*f*_FeOOH_ × *k*_OM_ × [OM]	a
5. Sulfate reduction	CH_2_O(NH_3_)_0.15_ + 0.5 SO_4_^2−^ + 0.65 H^+^ → 0.5 HS^−^ + CO_2_ + H_2_O + 0.15 NH_4_^+^	*f*_SO4_ × *k*_OM_ × [OM]	a
6. Methanogenesis	CH_2_O(NH_3_)_0.15_ + 0.15 H_2_O → 0.5 CH_4_ + 0.35 CO_2_ + 0.15 HCO_3_^−^ + 0.15 NH_4_^+^	*f*_OM_ × *k*_OM_ × [OM] × switch	a,c
Secondary redox reactions			
7. NH_4_^+^ oxidation by O_2_	NH_4_^+^ + 2 O_2_ + 2 HCO_3_^−^ → NO_3_^−^ + 2 CO_2_ + 3 H_2_O	*k*_7_ × [NH_4_^+^] × [O_2_]	
8. As(III) oxidation by O_2_	H_3_AsO_3_ + 0.5 O_2_ → HAsO_4_^2−^ + 2 H^+^	*k*_8_ × [O_2_] × [As(III)]	d
9. Fe^+2^ oxidation by O_2_	Fe^2+^ + 0.25 O_2_ + 2 HCO_3_^−^ → FeOOH + 2 CO_2_ + 0.5 H_2_O	*k*_9_ × [Fe^+2^] × [O_2_]	
10. FeS(s) oxidation by O_2_	FeS(s) + 2 O_2_ → SO_4_^2−^ + Fe^2+^	*k*_10_ × [FeS] × [O_2_]	
11. FeS_2_(s) oxidation by O_2_	FeS_2_(s) + 1.5 O_2_ + H_2_O → 2 SO_4_^2−^ + Fe^2+^ + 2 H^+^	*k*_11_ × [FeS_2_] × [O_2_]	
12. H_2_S oxidation by O_2_	H_2_S + 2 O_2_ + 2 HCO_3_^−^ → SO_4_^2−^ + 2 CO_2_ + 2 H_2_O	*k*_12_ × [H_2_S] × [O_2_]	
13. CH_4_ oxidation by O_2_	CH_4_ + 2 O_2_ → CO_2_ + 2 H_2_O	*k*_13_ × [CH_4_] × [O_2_]	
14. FeS(s) oxidation by As(V)	FeS(s) + 4 HAsO_4_^2−^ + 8 H^+^ → Fe^2+^ + SO_4_^2−^ + 4 H_3_AsO_3_	*k*_14_ × [FeS] × [HAsO_4_^2−^]	
15. FeS_2_(s) oxidation by As(V)	FeS_2_(s) + 7 HAsO_4_^2−^ + H_2_O + 12 H^+^ → Fe^2+^ + 2 SO_4_^2−^ +7 H_3_AsO_3_	*k*_15_ × [FeS_2_] × [HAsO_4_^2−^]	
16. H_2_S oxidation by As(V)	H_2_S + 4 HAsO_4_^2−^ + 6 H^+^ → SO_4_^2−^ + 4 H_3_AsO_3_	*k*_16_ × [H_2_S] × [HAsO_4_^2−^]	
17. H_2_S oxidation by FeOOH	H_2_S + 14 CO_2_ + 8 FeOOH + 2 H_2_O → 8 Fe^2+^ + SO_4_^2−^ +14 HCO_3_^−^	*k*_17_ × [H_2_S] × [FeOOH]	
18. CH_4_ oxidation by SO_4_^2−^	CH_4_ + SO_4_^2−^ + CO_2_ → H_2_S + 2 HCO_3_^−^	*k*_18_ × [CH_4_] × [SO_4_^2−^]	
Other kinetic processes			
19. Pyrite precipitation	FeS(s) + H_2_S → FeS_2_ + H_2_	*k*_19_ × [FeS] × [H_2_S]	e
20. Mercury methylation	CH_4_ + Hg^2+^ → CH_3_Hg^+^ + H^+^	*k*_20_ × *R*_5_ × ([HOHgSH] +[Hg(SH)_2_])	f
21. Mercury demethylation	CH_3_Hg^+^ + H^+^ → CH_4_ + Hg^2+^	*k*_21_ × [MeHg]_tot_	g

^a^Concentration of OM refers to either SOM or DOM, which were separately degraded in the model; *f*_*i*_ is the fraction of electrons consumed by the *i*th primary reduction half-reaction and *k*_OM_ is the rate constant for OM degradation. For all reactions, rate constants are given in Table 5

^b^Rate multiplied by acceleration term (accel) to account for faster biodegradation in presence of oxygen and nitrate

^c^Rate multiplied by switch function (switch) =1 when CH_4_(aq) concentration is below the solubility of CH_4_(g) and =0 when greater than the solubility limit

^d^Rate modeled as a function of total As(III) concentration

^e^Rate expression of Canavan et al. (2006) used for conversion of mackinawite (treated as an equilibrium species) to pyrite. The same rate expression was used for the conversion of FeS(aq), but supersaturation with respect to pyrite was required (SI = 14; see discussion in Rickard and Luther 2007)

^f^Rate equation includes term *R*_5_ (sulfate reduction rate) and a factor that calculates methylation rate based on the activity of sulfate-reducing bacteria and the total concentration of neutral mercury sulfide complexes

^g^Simplified reaction based on [MeHg]_tot_, which is the total (dimethylmercury plus methylmercury) concentration; model does not distinguish between products of oxidative or reductive demethylation processes as discussed in Ullrich et al. (2001)

**Table 5:** Parameter values for kinetic reactions (shown in Table 4)

Parameter	Value	Units	Reported range	Comment
Primary redox reactions				
*k*_SOM_	0.002	year^−1^	0.0003–0.02	a
*k*_DOM_	0.001	year^−1^	0.001	b
Accel	10		25	c
*f*_EA_	0–1			d
Limiting concentrations for electron acceptor species (*K*_EA_)				
*K*_O2_	10	µM	8–24	e
*K*_NO3_	10	µM	10–16	e
*K*_As(V)_	10	µM		f
*K*_FeOOH_	1,000	µM	800–1,000	e
*K*_SO4_	100	µM	100	c
Secondary redox reactions				
*k*_7_	10	µM^−1^ year^−1^	5–79	g
*k*_8_	10	µM^−1^ year^−1^		h
*k*_9_	10	µM^−1^ year^−1^	0.35–16,000	g
*k*_10_	1	µM^−1^ year^−1^	0.19–20	g
*k*_11_	1	µM^−1^ year^−1^		i
*k*_12_	100	µM^−1^ year^−1^	0.16–1,600	g
*k*_13_	10,000	µM^−1^ year^−1^	10,000	g
*k*_14_	1	µM^−1^ year^−1^		j
*k*_15_	1	µM^−1^ year^−1^		k
*k*_16_	1	µM^−1^ year^−1^		k
*k*_17_	0.01	µM^−1^ year^−1^	0.008–0.095	g
*k*_18_	1	µM^−1^ year^−1^	0.01–10,000	g
Other kinetic processes				
*k*_19_	3.3 × 10^−3^	µM^−1^ year^−1^		c
*k*_20_	4.2 × 10^3^	M M_SO4_^−1^ M_HgS_^−1^ year^−1^		l
*k*_21_	3 × 10^−4^	year^−1^	1–14 × 10^−4^	m

^a^Reported range for SOM from Arzayus and Canuel (2005), Canfield et al. (1993), Fossing et al. (2004), Hulthe et al. (1998), Wallmann et al. (2006)

^b^Reported range for DOM from Komada et al. (2004)

^c^Canavan et al. (2006)

^d^Calculated by the model

^e^Reported range from Canavan et al. (2006) (*K*_FeOOH_ approximated)

^f^Set equal to nitrate

^g^ Reported range from Canavan et al. (2006), which included the studies of Berg et al. (2003), Fossing et al. (2004), Van Cappellen and Wang (1995, 1996)

^h^Set equal to rate constant for Fe^2+^ oxidation (*k*_9_)

^i^Set equal to rate constant for FeS(s) oxidation (*k*_10_)

^j^Estimated from reported rate for arsenate reduction by dissolved sulfide of Rochette et al. (2000)

^k^Set equal to rate constant for FeS(s) oxidation (*k*_14_)

^l^See text for explanation of terms

^m^Reported range from Hintelmann et al. (2000), Kim et al. (2004), Marvin-Dipasquale and Oremland (1998), Drott et al. (2008)

Arsenate reduction and secondary arsenite oxidation reactions were added to the suite of reactions compiled from prior studies (Table 4). Arsenate reduction was assumed to precede reduction of ferrous to ferric iron in the sequence of terminal EA based on a larger (more negative) change in the Gibbs free energy of the half-reaction for arsenate reduction compared with goethite reduction. Values for the limiting concentrations and secondary reaction rates for iron sulfide oxidation by arsenate were estimated (Table 5) from rates reported for arsenate reduction by sulfide (Rochette et al. 2000).

The rate of methylmercury formation was calculated using the following equation developed by Gilmour et al. (2008):

2 $$ {\text{rate}}_{{{\text{CH}}_{ 3} {\text{Hg}}^{ + } }} = \lambda \times \left[ {\text{cells}} \right] \times \left[ {{\text{C}}_{1} } \right] \times \left( {\left[ {\text{OHHgSH}} \right] + \left[ {{\text{Hg}}\left( {\text{SH}} \right)_{2} } \right]} \right) $$

where the parameter *λ* represents the fraction of sulfate-reducing bacteria that methylate mercury at the same rate as *Desulfobulbus propionicus* (a value of 0.2 was used both in Gilmour et al. (2008) and here); [cells] is the number of cells of sulfate-reducing bacteria calculated from the model-determined sulfate reduction rate (M_SO4_ year^−1^, Table 4) and a reported cell density corresponding to this rate (1.63 × 10^12^ cells year M_SO4_^−1^) (King et al. 2000); the methylation rate parameter [*C*_1_] was assumed to be 8.54 × 10^−8^ M_MeHg_ L cell^−1^ M_ΣHgSneutral_^−1^ year^−1^ (Gilmour et al. 2008); and the total concentration of dissolved neutral mercury sulfide complexes ([OHHgSH] + [Hg(SH)_2_]) (M_HgS_ L^−1^) was computed by PHREEQC. The equivalent expression re-written as Eq. 20 in Table 4 by combining terms in Eq. 2 is:

3 $$ {\text{rate}}_{{{\text{CH}}_{ 3} {\text{Hg}}^{ + } }} = k_{20} \times R_{5} \times \left( {\left[ {\text{HOHgSH}} \right] + \left[ {{\text{Hg}}\left( {\text{SH}} \right)_{2} } \right]} \right) $$

where the rate constant [*k*_20_] is 4.2 × 10^3^ M M_SO4_^−1^ M_HgS_^−1^ year^−1^ (M_HgS_ represents the total concentration of the two neutral mercury sulfide complexes) (Table 5), *R*_5_ is the rate of sulfate reduction (Table 4), and [HOHgSH] and [Hg(SH)_2_] are the concentrations of the two neutral mercury sulfide aqueous complexes included in the model (“Appendix 2”). Demethylation was modeled by using a first-order rate constant with respect to the total MeHg concentration (dimethylmercury plus monomethylmercury species).

Performance of the model in determining mercury methylation was evaluated by simulating the controlled microcosm experiments of Johnson et al. (2010). Concentrations of methylmercury were calculated by the model based on the chemical description of the sediment and porewater from Johnson et al. (2010), the estimated organic carbon biodegradation rate from that study, and methylation/demethylation implemented in the PHREEQC model with the parameters given in Table 5. The model predicted a total MeHg concentration in the sediment after a 120-day simulation period of 230 pg/g, which is equivalent to 0.07 % of the total mercury concentration. This concentration compares favorably with reported concentrations of 215–232 pg/g (equivalent to 0.06–0.08 % of total mercury present as methylmercury) at the deepest measurement point in the sediment microcosms (0.03 m) (Johnson et al. 2010).

## Results

Model results were assessed for the following: (1) the evolution of solid-phase contaminant sequestration, either by mineral precipitation or surface adsorption, in the subaqueous sediment cap (reported as total moles of solid/L of water to facilitate direct comparison with the aqueous concentrations), (2) the flux of contaminants to surface water over time, and (3) geochemical environments where capping would be most effective based on model outcomes. The latter criterion was assessed by comparing simulated aqueous and solid-phase concentrations with water and sediment quality screening values that might be employed during remedial design. Table 3 summarizes the model scenarios.

### Arsenic

**Fig. 2:**
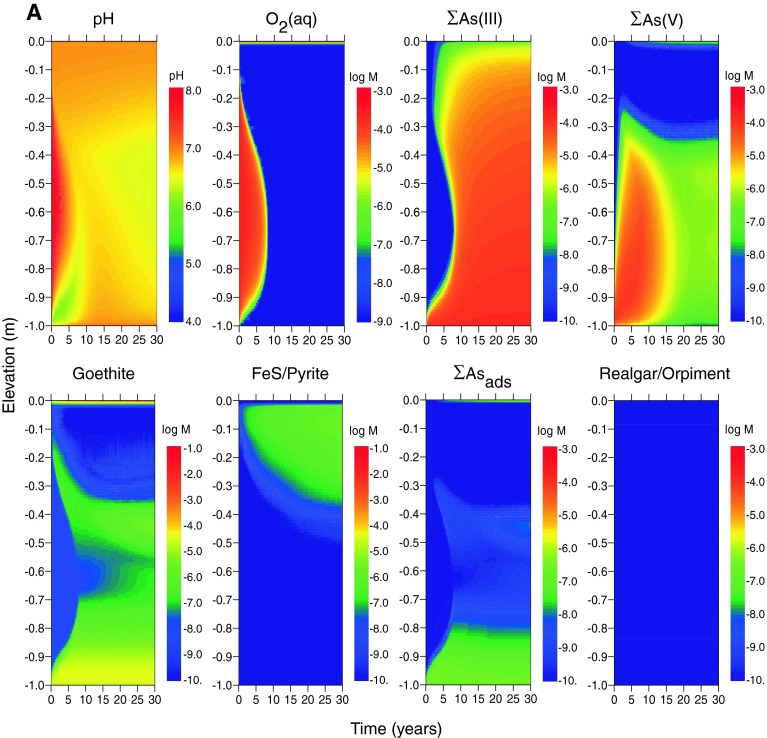

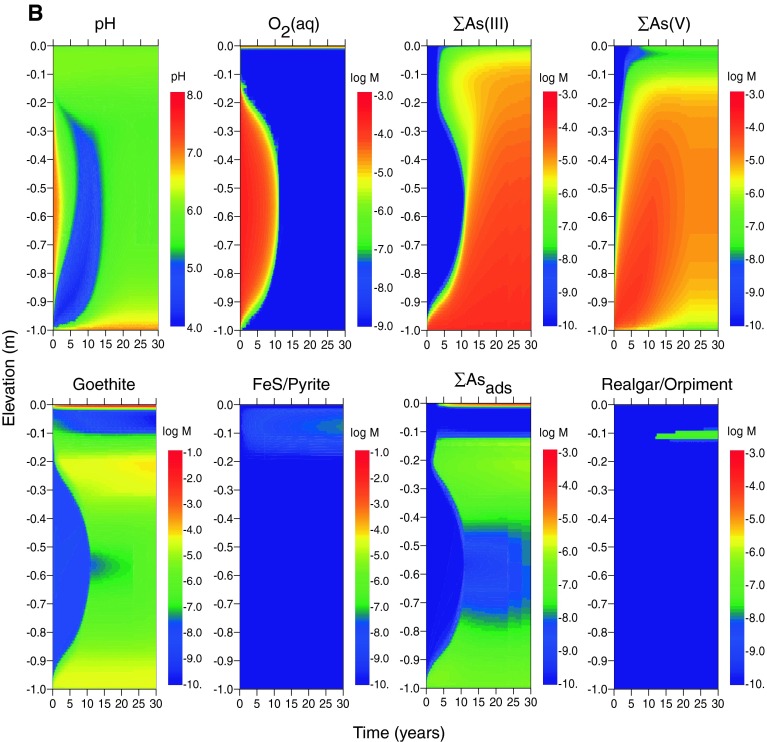
Concentrations of Fe and As mineral and dissolved species (in log moles/L of water) as a function of depth and time in the sediment cap for the case of diffusion only, log [As]_tot_ = −3.9 and log [Hg]_tot_ = −7.3 in influent porewater, and the following environments. **a** Estuarine (scenario E1), and **b** freshwater (scenario F1). *Rows* 1 and 3 (*left*–*right*): pH; dissolved oxygen; total dissolved As(III); total dissolved As(V). *Rows* 2 and 4 (*left*–*right*): goethite; mackinawite and pyrite; As adsorbed to goethite; realgar and/or orpiment

The simulated evolution of cap mineralogy and porewater geochemistry for the case of estuarine boundary conditions and diffusion-only porewater flux (scenario E1) are shown as a function of time and depth in Fig. 2a. The first 30 years of simulation results are illustrated, after which steady-state conditions are established. After the first decade, pH (~6.5–6.7) and dissolved oxygen (DO ~ 10^−8^ M) are constant within the sediment cap, with the exception of a thin oxidized zone (~0.02 m) at the sediment–water interface. Dissolved arsenic is highest at the base of the cap from porewater influx and decreases toward the sediment–water interface. The concentration of As(V) decreases with time in the lower half of the cap and dissolved arsenic is dominated by As(III) at steady state. Goethite precipitates at the oxidized sediment–water interface as a result of dissolved Fe(II) oxidation by diffusion of oxygen from surface water. Goethite also precipitates at the base of the cap, where more oxidized porewater (due to surface water initially entrained in the cap) mixes with influent, anaerobic porewater containing Fe(II) (Table 1). Goethite precipitation near the base of the cap (0.9–1.0 m depth) decreases upward as Fe(II) introduced by porewater is depleted. A zone of reduced iron sulfide minerals (mackinawite and pyrite) forms between ~0.02 and 0.3 m depth from the reduction of sulfate supplied by diffusion of seawater. These minerals replace goethite with time as the primary iron solid phases in the upper part of the cap. However, arsenic sulfide minerals do not form under these conditions. For the diffusion-only scenario, the total dissolved arsenic concentration in equilibrium with goethite in the top 0.1 m at the end of the model simulation (50 years) is 10^−5.5^ M (0.26 mg/L) (Table 6).

**Table 6:** Depth-averaged results (from 0 to 0.1 m) for cap simulations after 50 years

Model scenario^a^	Input		As (aq)_tot_	As (tot)^b^	Hg (aq)_tot_	Hg (tot)^b^	MeHg (aq)_tot_	MeHg (tot)^b^
	As	Hg						
	Log M	Log M	Log M	Log (mol g^−1^)	Log M	Log (mol g^−1^)	Log M	Log (mol g^−1^)
Estuarine								
E1: diff, high conc.	−3.9	−7.3	−5.5	−9.0	−11.2	−14.5	−13.1	−16.3
E2: diff + advection	−3.9	−7.3	−4.5	−8.1	−10.6	−13.8	−12.4	−15.5
E3: diff + goethite	−3.9	−7.3	−6.8	−9.5	−11.1	−12.9	−12.6	−15.0
E4: diff, low conc	−5.2	−8.6	−6.8	−10.3	−11.6	−14.9	−13.5	−16.7
Freshwater								
F1: diff, high conc.	−3.9	−7.3	−5.5	−8.4	−11.0	−12.6	−13.5	−15.8
F2: diff + advection	−3.9	−7.3	−4.5	−7.9	−10.6	−12.1	−13.1	−15.1
F3: diff + goethite	−3.9	−7.3	−7.5	−9.9	−12.3	−13.2	−14.7	−16.2
F4: diff, low conc.	−5.2	−8.6	−6.8	−8.9	−11.2	−12.7	−13.7	−16.0

^a^See Table 3 for scenario conditions

^b^Total solid-phase concentration in log (mol g^−1^ sediment)

In the freshwater scenario (F1) (Fig. 2b), steady-state concentrations are not established in most of the cap until after ~20 years. Similar to the estuarine case, reduced conditions are established throughout the cap, but the steady-state pH is lower (~6) in the freshwater than in the estuarine simulation. Dissolved arsenic is dominated by As(III) at steady state, but As(V) is elevated from the base of the cap to ~0.15 m below the surface in the habitat layer. Arsenic sequestration by solid phases in the freshwater scenario is distributed between adsorption by goethite and a zone of realgar (AsS) precipitation at 0.1–0.15 m depth. Greater quantities of both minerals are precipitated in the upper part of the cap compared with the estuarine scenario. For goethite, this outcome is directly related to lower total sulfur concentration, and therefore lower sulfide levels, in freshwater. Lower total sulfur affects goethite precipitation in two ways: (1) no pyrite or mackinawite forms in freshwater, which increases the concentration of dissolved Fe(II) available for oxidation by surface water, and (2) less sulfide is available to reduce Fe(III) (and thereby dissolve goethite). As a result, an interval of goethite forms between ~0.15 and 0.3 m below the surface in addition to zones above the base of the cap and at the sediment–water interface (Fig. 2b). For realgar, the effect of lower sulfide in the freshwater scenario is to decrease the concentration of As-sulfide complexes and thus increase the concentration of dissolved As(OH)_3_°, exceeding reaglar solubility. Realgar precipitation, however, does not greatly decrease dissolved arsenic concentrations in the cap because of its higher equilibrium solubility compared with other sulfide minerals. At 50 years, the total dissolved arsenic concentration at the top of the cap is predicted to be the same (10^−5.5^ M) as for the estuarine scenario (Table 6), although the distribution of arsenic species is different throughout the simulation period.

**Fig. 3:**
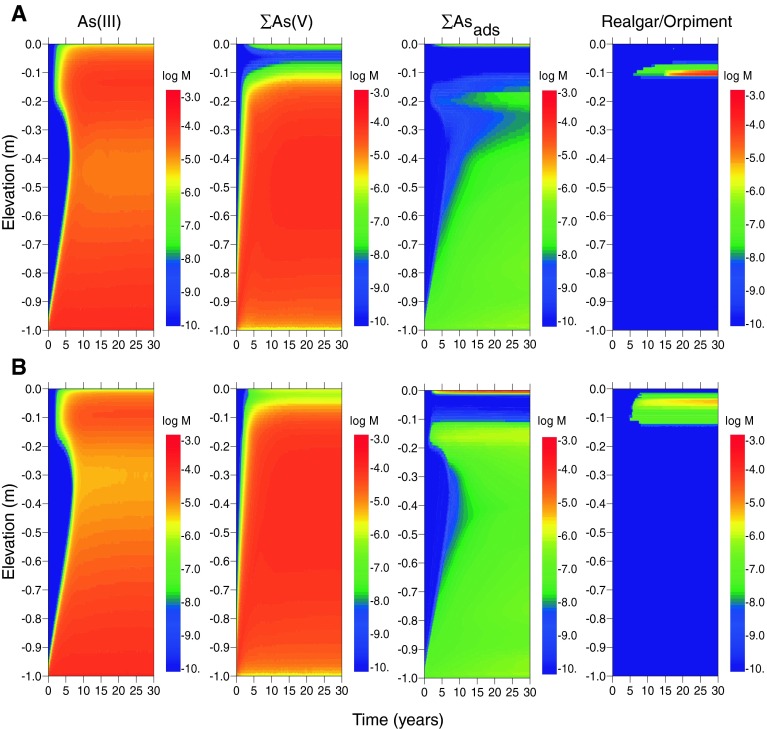
Concentrations of As mineral and dissolved species (in log moles/L of water) as a function of depth and time in the sediment cap for the case of advecting groundwater, log [As]_tot_ = −3.9, log [Hg]_tot_ = −7.3 in influent porewater, and the following environments. **a** Estuarine (scenario E2), and **b** freshwater (scenario F2). *Rows* 1 and 2 (*left*–*right*): total dissolved As(III); total dissolved As(V); As adsorbed to goethite; realgar and/or orpiment

Model outcomes for arsenic with advective transport in addition to diffusion in both estuarine (scenario E2) and freshwater (scenario F2) systems are shown in Fig. 3a and b, respectively. One effect of advection is to decrease the time required for the establishment of steady-state dissolved profiles to a period of <10 years for most species. A second is to introduce greater quantities of arsenic into the cap and therefore generate higher dissolved arsenic concentrations throughout the cap and near the sediment–water interface. As shown in Table 6, dissolved arsenic concentrations at the top of the cap are ~10 times higher than in the diffusion-only scenarios. The introduction of higher arsenic concentrations into the habitat layer (where sulfide is generated from the reduction of sulfate) causes more precipitation of realgar where the upward-propagating arsenic flux meets the downward-propagating sulfide flux. Arsenic is also sequestered in higher amounts by the thin goethite layer at the sediment–water interface. The advection scenarios highlight the competition between arsenic sequestration by adsorption to goethite and precipitation of arsenic sulfide minerals.

In order to examine model sensitivity to iron concentration in the cap and to contaminant flux from upwelling porewater, additional scenarios were examined for the case of an initial coating of goethite on quartz in the sand cap (scenarios E3 and F3) (Knapp et al. 2002; Kent and Fox 2004) and for lower influent contaminant concentrations (scenarios E4 and F4). As shown in Table 6, the effect of goethite coatings is to reduce the mass flux of arsenic through the cap and thus the dissolved and solid concentrations at the top of the cap. Lower influent porewater arsenic concentrations result in concentrations at the top of the cap similar to those observed with more goethite coatings because attenuation is primarily controlled by arsenic adsorption to goethite in these scenarios.

### Mercury

**Fig. 4:**
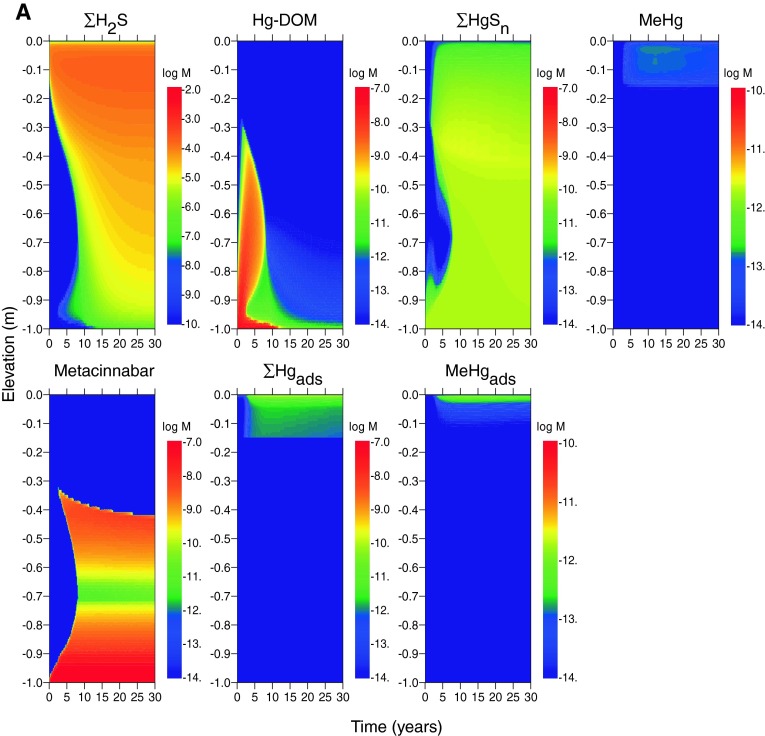

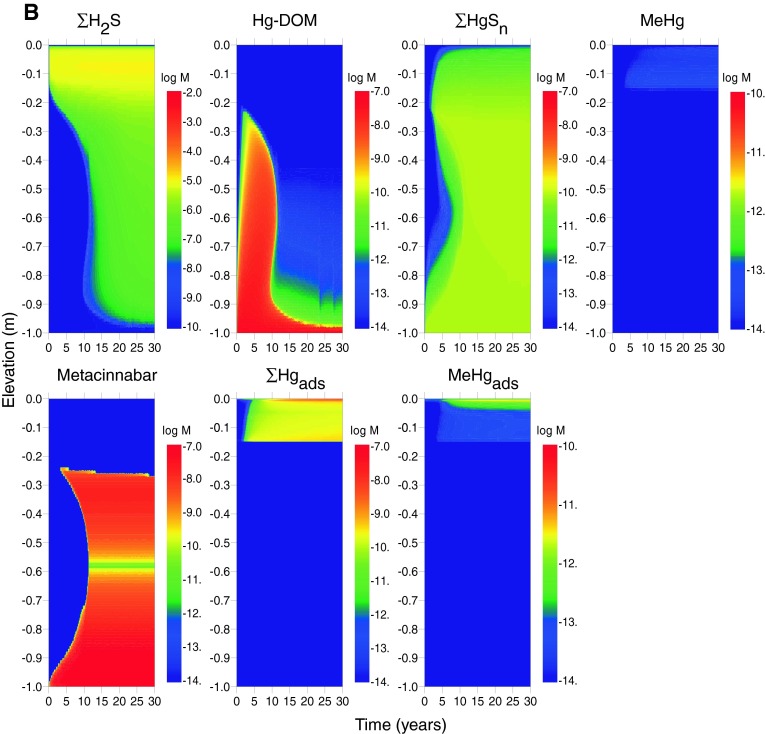
Concentrations of S and Hg mineral and dissolved species (in log moles/L of water) as a function of depth and time in the sediment cap for the case of diffusion-only, log [As]_tot_ = −3.9, log [Hg]_tot_ = −7.3 in influent porewater, and the following environments. **a** Estuarine (scenario E1), and **b** freshwater (scenario F1). *Row* 1 (*left*–*right*): total dissolved sulfide (all species); Hg-DOM complexes; total dissolved Hg-sulfide complexes; dissolved methylmercury (mono- and di-methyl). *Row* 2 (*left*–*right*): metacinnabar (HgS); total mercury adsorbed to SOM, total MeHg adsorbed to SOM

For the estuarine, diffusion-only sediment cap (scenario E1) (Fig. 4a), dissolved mercury concentrations are highest at early times and near the base of the cap as Hg complexed to DOM. Mercury speciation changes to complexation with dissolved sulfide within the first 5–10 years as reduced conditions are established and sulfate reduction produces dissolved sulfide. The primary solid-phase sequestration mechanism for mercury is precipitation as metacinnabar [HgS(s)] once its solubility is exceeded with sufficient sulfide production. Metacinnabar precipitation initially occurs in two areas of the cap: (1) near the base where a metacinnabar front propagates upward and (2) a depth between 0.4 and 0.6 m where a metacinnabar front propagates downward. Dissolved sulfide concentrations build over time in the upper third of the cap, which results in higher concentrations of dissolved Hg–sulfide complexes and shifts the depth of the precipitation front for metacinnabar downward. In the upper habitat layer (0–0.15 m), Hg is attenuated in the cap by sorption to SOM.

Dissolved and solid-phase mercury profiles in freshwater (scenario F1) are generally similar to the estuarine scenario (E1) except for (1) the occurrence at a shallower depth of the metacinnabar precipitation front and (2) greater mercury complexation to SOM in the top 0.15 m of the cap (Fig. 4b). Differences in mercury speciation and partitioning can be attributed to less total sulfur, and therefore less sulfide, in freshwater versus estuarine systems. In the freshwater case, more time is required for sulfide concentrations to build to levels where metacinnabar precipitates in the cap. Thus, the concentration of Hg–sulfide complexes is lower at the top of the cap, resulting in a shallower depth of precipitation of metacinnabar and greater partitioning to SOM. The steady-state total dissolved mercury concentration in the top 0.1 m in the estuarine sediment cap is slightly less than in the freshwater cap (Table 6) because metacinnabar precipitation occurs closer to the sediment–water interface in the latter case.

**Fig. 5:**
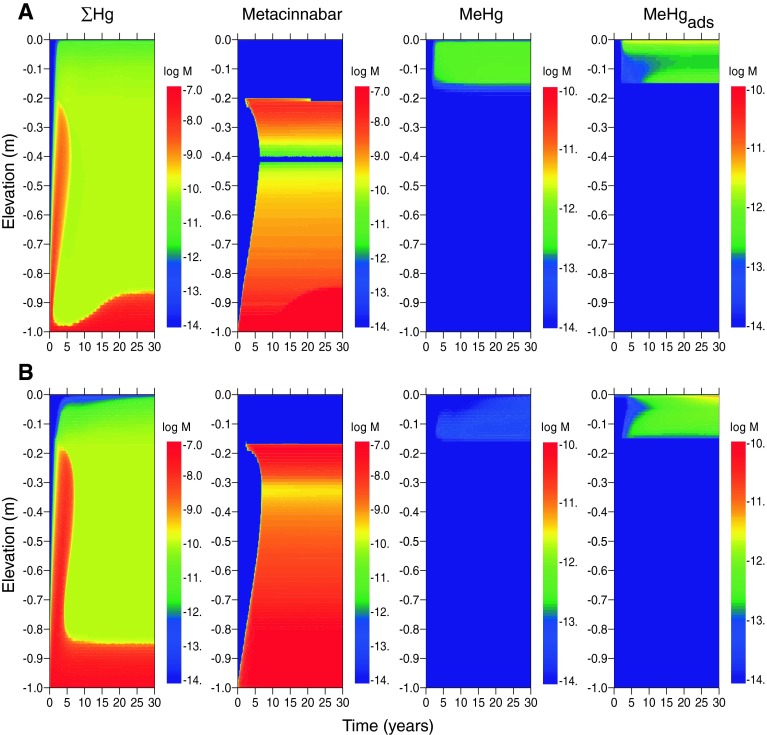
Concentrations of Hg mineral and dissolved species (in log moles/L of water) as a function of depth and time in the sediment cap for the case of advecting groundwater, log [As]_tot_ = −3.9, log [Hg]_tot_ = −7.3 in influent porewater, and the following environments. **a** Estuarine (scenario E2), and **b** freshwater (scenario F2). *Rows* 1 and 2 (*left*–*right*): total dissolved Hg; metacinnabar (HgS); total dissolved methylmercury; methylmercury adsorbed to SOM

The effect of porewater advection (scenarios E2 and F2) is to shift the metacinnabar precipitation front upwards in the cap, which increases dissolved mercury concentrations at shallower depths (Fig. 5a, b). For estuarine systems, steady-state dissolved concentrations in the top 0.1 m of the cap increase from 10^−11.2^ M (1.2 ng/L) to 10^−10.6^ M (5.0 ng/L) (Table 6). For freshwater, steady-state concentrations increase from 10^−11.0^ M (1.9 ng/L) to 10^−10.6^ M (5.5 ng/L). These concentrations are generally within the range reported for estuarine surface water (10^−9.8^–10^−12.4^ M) (Fitzgerald et al. 2007). Unlike arsenic, an initial coating of goethite in the cap causes little change in dissolved mercury concentrations because mercury adsorption to iron oxide minerals was not included in the model and is expected to be less important than sorption to OM (Feyte et al. 2010).

### Methylmercury

Methylmercury formation is predicted to occur within the upper 0.15 m of the cap in both estuarine and freshwater scenarios (E1 and F1) (Fig. 4a, b). Dissolved methylmercury concentrations are approximately 1–4 % of the total mercury, which is typical for sediment environments (Ullrich et al. 2001). The primary attenuation mechanisms for methylmercury are demethylation and adsorption of methylmercury to SOM. In these model scenarios, the mass of adsorbed methylmercury generally exceeds the dissolved mass (Figs. 4, 5). Dissolved methylmercury concentrations are slightly lower at the top of the freshwater cap than the estuarine cap because the concentrations of neutral mercury sulfide complexes available for methylation are lower (owing to lower total sulfur) (Table 6). In contrast, methylmercury concentrations in sediment are higher in fresh than salt water because concentrations of dissolved methylmercury-sulfide complexes are lower and more methylmercury is available for sorption to SOM.

Advective transport increases the steady-state concentration of methylmercury in both estuarine and freshwater systems (Fig. 5). The highest dissolved concentrations occur in the estuarine sediment cap with advection, and the highest sediment-bound methylmercury levels occur in freshwater. Concentrations are predicted to be lower than those typically reported for contaminated sediments (Bloom et al. 1999) and are within the range of estuarine and riverine systems (Conaway et al. 2003; Marvin-DiPasquale et al. 2000; Marvin-Dipasquale and Oremland 1998; Goulet et al. 2007; Fitzgerald et al. 2007).

### Comparison with Screening Levels for Water and Sediment Quality

**Fig. 6:**
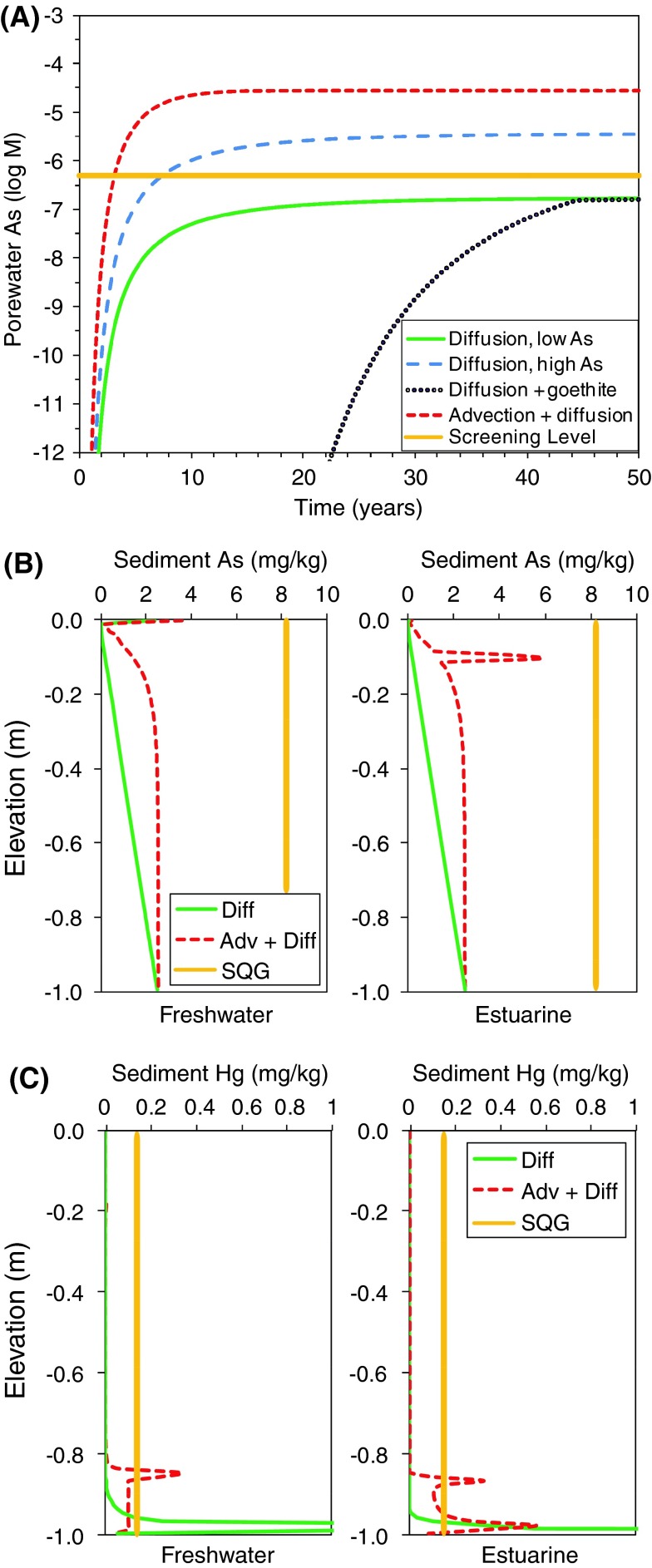
Comparison of predictions from model scenarios (Table 3) to water and sediment quality screening levels at 50 years. **a** Dissolved arsenic concentrations compared with CCC (*yellow line*) for estuarine model results. *Solid green* diffusion, low concentration (E4), *dashed blue* diffusion, high concentration (E1), *stippled black* diffusion with goethite coating (E3), *dashed red* advection + diffusion, high concentration (E2). Comparison of **b** arsenic and **c** mercury sediment concentrations in freshwater and estuarine conditions with SQG (SQG = *yellow line*) in diffusion-only (*solid green*) and diffusion plus 0.1-m/year advection (*dashed red*) scenarios

Model outcomes at 50 years were compared with two quality criteria: criterion continuous concentration (CCC) for dissolved contaminants in saltwater and freshwater (USEPA 2009), and sediment quality guidelines (SQG) using the effects range-low (ERL) values from (NOAA 1999). The CCC is an estimate of the highest concentration of a material in surface water to which an aquatic community can be exposed indefinitely without resulting in an unacceptable effect. The ERL guidelines are intended to represent sediment concentrations of a contaminant below which adverse effects on biota rarely occur and are derived from the 10th percentile values of compiled studies below which adverse effects occurred. Comparison of model outcomes (Table 6) showed that for all high influent arsenic concentration scenarios, dissolved arsenic concentrations at the top of the cap (depth-averaged from 0 to 0.1 m) exceeded CCC levels for both freshwater (10^−5.7^ M) and saltwater (10^−6.3^ M) (Fig. 6a). When influent arsenic concentration was reduced (20 times lower, scenarios E4 and F4), depth-averaged concentrations in both estuarine and freshwater sediment caps were below CCC levels (36, and 150 µg/L, respectively). In the presence of iron oxyhydroxides (as goethite) on quartz (scenario E3), concentrations approached but did not exceed the CCC in the top 0.1 m of the cap (Fig. 6a). Dissolved mercury concentrations (Table 6) were several orders-of-magnitude below the CCC for all scenarios (data not shown).

Sediment arsenic concentrations were below ERL levels for both high and low concentration scenarios (Fig. 6b). For mercury, sediment ERL concentrations were exceeded only within the lower 0.2 m of the cap where high concentrations of HgS(s) were precipitated and were below screening levels at the top (Fig. 6c). Methylmercury concentrations in sediments (not shown) are predicted to be below regulatory screening levels.

## Discussion

### Factors Controlling Cap Effectiveness

The analysis of mineralogical and porewater changes within a sediment cap by reactive transport modeling highlights primary factors that control the migration of dissolved contaminants from the cap into surface waters. An important observation from this analysis is that different but interrelated factors are responsible for arsenic or mercury attenuation in the cap. From the standpoint of (bio)geochemical reactions, controlling processes are (1) the concentration of DOM and SOM in the system, which influences (a) the amount of iron and sulfate reduction, and (b) the extent of mercury and methylmercury complexation with DOM in solution and sorption to SOM in sediments. (2) The total amount of iron in the system from either sediment Fe(III) oxyhydroxides or groundwater influx of dissolved Fe(II). Total iron controls (a) the precipitation of Fe(III)-oxide minerals and thus arsenic sorption in more oxidized zones, and (b) the concentration of dissolved Fe(II) in more reduced zones. (3) The total amount of sulfur in the system, examined in this study by the difference between typical estuarine and freshwater systems. Total sulfur determines the maximum amount of sulfate reduction and thus, (a) the precipitation of iron, arsenic, and mercury sulfide phases and (b) the concentration of aqueous sulfide complexes. In addition, the amount of porewater advection, compared with diffusion only, will determine the flux of contaminants moving through the cap and thus the ability of (bio)geochemical processes to attenuate them.

In estuarine systems, total sulfur is in excess because of diffusion of sulfate from overlying surface water and from porewater influx of sulfate (Table 1). Because dissolved sulfide is generally higher throughout the cap than in the freshwater case, iron sulfide minerals (mackinawite and pyrite) form in the upper part of the cap, and the formation of iron oxide minerals (modeled here as goethite) is less important and limited to the bottom of the cap. Therefore, arsenic sorption to iron oxides is lower overall. No precipitation of arsenic sulfide minerals (realgar or orpiment) is observed because of the higher solubility of arsenic sulfides compared with iron sulfides or metacinnabar at the pH of these systems. In the freshwater simulations with lower total sulfur, iron sulfide minerals do not form, a small amount of realgar precipitates within the top 0.15 m, and goethite is stable below the top 0.15 m. Arsenic sorption to goethite occurs within the cap and at the oxidized sediment–water interface. However, dissolved arsenic concentrations in the top 0.1 m are similar to the estuarine case because the sorption capacity of iron oxides is the limiting factor for arsenic attenuation. Thus, dissolved arsenic flux from the cap for scenarios with high porewater concentration or with advection is higher than water quality screening levels because of insufficient attenuation by sorption to iron oxides and higher solubility of arsenic sulfide minerals compared with mercury sulfide.

In real systems, metastable Fe(III)-oxide phases such as ferrihydrite, or metastable Fe(II, III) oxides such as green rust-type phases, may form instead of the thermodynamically stable phases of goethite and magnetite used in this model. These metastable iron phases generally have higher surface areas than their stable counterparts and thus may be more effective sorbents of arsenic. On timescales longer than those modeled, continued precipitation of iron oxides may create a natural sorption barrier for arsenic (Jung et al. 2009), but only if the rate of oxide precipitation and generation of reactive surface sites exceeds the rate that arsenic is introduced into the system. Because sorption is generally more effective for arsenic attenuation than sulfide precipitation, cap performance depends on arsenic concentration and flux through the cap, the concentration and type of iron oxide minerals and their sorption sites, and porewater pH within the cap.

Mercury and methylmercury concentrations are predicted to remain below water and sediment quality criteria levels in both estuarine and freshwater systems once sulfide concentrations increase after the first 5–10 years because of precipitation of metacinnabar in the lower parts of the cap. Due to the very low solubility of mercury sulfide minerals, equilibrium dissolved mercury concentrations are low. In the upper part of the cap without metacinnabar, predicted methylmercury concentrations are slightly higher in the estuarine than the freshwater setting because of higher rates of sulfate reduction. The distribution of mercury and methylmercury between aqueous and solid phases in this zone is controlled by sorption to SOM, aqueous complexation with DOM, and aqueous complexation with sulfide. Therefore, the amount and distribution of OM are the primary controlling factors for mercury and methylmercury flux from the top of the cap. The concentration of thiol sites associated with SOM is the primary factor for mercury attenuation by sorption in sediments, which competes with sulfur binding sites in DOM as a possible mode of mercury transport in the aqueous phase.

### Model Uncertainties

Important sources of uncertainty in any chemical model are the values of equilibrium constants for reactions assumed to reach thermodynamic equilibrium, and in the rate constants for reactions assumed to be controlled by kinetic processes. For the majority of equilbrium reactions in the LLNL database and those that were added in this study (see “Appendices 1 and 2”), equilibrium constants are generally accepted and robust, with the following notable exceptions. As discussed above, there is a large uncertainty regarding equilibrium constants for aqueous mercury complexation (Skyllberg 2008), which affects model-predicted methylmercury levels (Drott et al. 2007). There is considerable uncertainty regarding both the identities and stability constants of arsenic and mercury mono- and poly-sulfide complexes, which may be important in high sulfide systems where elemental sulfur is stable (Rickard and Luther, 2006). Although mercury and mercury-DOM complexes may adsorb to quartz and iron oxides (Tiffreau et al. 1995; Backstrom et al. 2003), sorption to oxides is likely secondary to complexation with dissolved sulfide and DOM, and sorption to SOM, and therefore was not included. Another simplifying assumption was the use of an average diffusion coefficient for all aqueous species, which neglects the effects of porosity and tortuosity on the diffusion rates of specific ions or complexes. A greater source of uncertainty, however, probably results from the kinetic model and rate constants selected for abiotic and biotic chemical reactions. The mechanisms and rates of mercury methylation and demethylation are complex because (1) iron-reducing bacteria may contribute to methylmercury concentrations (particularly in freshwater caps; see Fitzgerald et al. 2007; Fleming et al. 2006), and (2) methylmercury production and degradation depends on temperature, pH, and other environmental variables (Ullrich et al. 2001). Uncertainties in these model parameters highlight the need for site-specific concentrations and rates in order to model remediation scenarios at contaminated sites.

Physical processes not included in the model may additionally affect contaminant distribution and fate within the cap. For example, this study did not explicitly include processes such as consolidation, bioturbation, bioirrigation, and sedimentation. Consolidation can temporarily increase the porewater flux through the cap, particularly at early times, thereby requiring a thicker cap to maintain low contaminant concentrations at the cap-water interface. Also, bioturbation and bioirrigation at the cap surface can affect the concentrations of both arsenic and mercury by creating a more diffuse zone of oxygenated conditions within the bioturbated layer, which could shift the sulfate/sulfide redox boundary deeper into the sediment (Dueri et al. 2003; Benoit et al. 2006). Inclusion of bioturbation and bioirrigation would likely increase the zone of Fe(III) oxide precipitation at the sediment–water interface, and thus the amount of iron oxide available for arsenic adsorption. The impact on arsenic breakthrough over long timescales would depend on the amount of Fe(II) available for oxidation and the arsenic flux. For mercury, greater introduction of oxygen into the habitat zone may decrease methylmercury production by slowing the rate of sulfate and/or iron reduction (Benoit et al. 2006). Finally, uncertainty is introduced by the imposed boundary conditions. For example, the constant concentration boundary conditions employed in this study give rise to sharp fronts of metacinnabar and goethite precipitation near the base of the cap. These sharp fronts are model artifacts that occur because of the prescribed boundary conditions. In reality, dissolved species may also migrate to some extent into the underlying contaminated sediment, thereby shifting the zone of precipitation downward.

### Cap Design Implications

The sediment cap simulations illustrate the fate of arsenic and mercury in various contaminated sediment–water environment scenarios and highlight some of the key factors to be considered for cap design. Unamended subaqueous sand caps are not expected to be effective for reducing porewater dissolved arsenic concentrations below ecological screening levels when concentrations in upwelling porewater are high (Fig. 6a). As shown in Fig. 6b, there is a potential over time for the habitat layer of the sediment cap to become contaminated in excess of SQG by either adsorption to iron oxides or precipitation of arsenic sulfides under sulfate-reducing conditions. The former is expected to be a feature of the uppermost few millimeters of the cap near the sediment–water interface where exposure to oxygenated water promotes formation of iron oxides. Arsenic sorption may be enhanced if the rate of ferrihydrite or goethite precipitation exceeds the arsenic flux into the cap, or if there is a pre-existing pool of iron oxyhydroxides in the sediment cap material (scenarios E3 and E4, respectively). Precipitation of arsenic sulfides, on the other hand, is more likely in estuarine/marine porewater environments where the downward diffusive flux of sulfate and the upward flux of arsenic provide dissolved reactants and bacterial sulfate reduction promotes the precipitation of solid-phase sulfides. The depth and extent of the zone of accumulation depends on the porewater upwelling velocity and the competitive precipitation of iron sulfide minerals. Due to the modest solubility of arsenic sulfides, they may occur closer to the top of the active sulfate-reducing zone where dissolved sulfide concentrations are greatest (Fig. 6b).

In contrast, capping with unamended sand is a potentially viable option for reducing mercury flux under all of the conditions modeled. The effectiveness is a function of (1) the rate of sulfate reduction, (2) porewater upwelling rates, and (3) cap thickness. As shown in Fig. 6c, mercury contamination is restricted to the base of the cap due to precipitation of insoluble mercury sulfide. Therefore, cap thickness appears to be a key design parameter for preventing mercury contamination of the habitat layer for a given flow regime because the upper depth limit of mercury accumulation is enhanced by porewater advection. The minimum thickness will need to be greater than the depth of the habitat layer in order to create a zone of separation between ecological receptors and mercury accumulation (Simpson et al. 2002). For the range of conditions examined in our study, the maximum thickness is expected to be <1 m, which is consistent with field studies (Moo-Young et al. 2001). The effectiveness of a sand cap for limiting mercury exposure to biota will depend on a number of site-specific physical, hydrologic, and biogeochemical factors that can be examined with reactive transport modeling as illustrated here.

## Conclusions

Reactive transport simulations show that the effectiveness of sand caps for remediating arsenic and mercury in subaqueous sediments, as measured by solid and aqueous-phase concentrations in the habitat layer, depends on (1) the rate of biologically mediated sulfate reduction, (2) dissolved contaminant concentrations and flux, (3) porewater advection rates, and (4) cap thickness. Arsenic attenuation depends primarily on pH-dependent sorption by iron oxide minerals and secondarily on arsenic sulfide precipitation. Therefore, prediction of cap performance with time depends on arsenic concentration and flux through the cap, the concentration and type of iron oxide mineral sorption sites, and pH. The primary controlling factor for mercury attenuation within the cap is mercury sulfide precipitation. In the habitat layer, however, attenuation of mercury and methylmercury are controlled by the amount and distribution of DOM and SOM, and in particular the concentration of thiol (–SH) groups associated with OM. Unamended caps are expected to be more effective for mercury attenuation than for arsenic attenuation in estuarine settings because of the lower solubility of mercury sulfide solid phases compared with arsenic sulfide phases. Dissolved and solid-phase contaminant concentrations are higher in cases where advective transport is important because introduction of additional contaminant mass can overwhelm attenuation mechanisms, particularly for arsenic sorption to iron oxides. The depth of mercury sulfide precipitation relative to the sediment–water interface can be partially controlled by changing cap thickness. The minimum thickness to reduce exposure to ecological receptors will be the depth of the habitat layer; however, the maximum required thickness may be as little as one meter. Site-specific quantification of the potential impact of biota on organic carbon is an important consideration during cap design, as are other physical processes such as compaction, sedimentation, bioturbation, and bioirrigation that influence the depth of the redox front below the sediment–water interface. Given the complex interplay between chemical, physical, and biological processes, reactive transport modeling provides a quantitative framework that can guide site-specific cap design, as well as lend insight into coupled physical-biogeochemical processes in subaqueous sediment systems.

## Appendix 1

See Table 7.

**Table 7:** Thermodynamic equilibrium constants for arsenic added to default LLNL database

Reaction	Log K^a^	Reference^b^
Aqueous As(III) species		
H_2_AsO_3_^−^ + H^+^ = H_3_AsO_3_	9.17	1
H_2_AsO_3_^−^ = HAsO_3_^−2^ + H^+^	−14.06	1
H_2_AsO_3_^−^ = AsO_3_^−3^ + 2 H^+^	−29.05	1
3 H_2_AsO_3_^−^ + 6 HS^−^ + 8 H^+^ = H_2_As_3_S_6_^−^ + 9 H_2_O	99.8	1
H_2_AsO_3_^−^ + 2 HS^−^ + 2 H^+^ = H_2_AsS_2_O^−^ + 2 H_2_O	27.2	1
Na^+^ + H_2_AsO_3_^−^ = NaH_2_AsO_3_	0.25	2
Ca^+2^ + H_2_AsO_3_^−^ = CaH_2_AsO_3_^+^	1.806	2
Fe^+3^ + H_2_AsO_3_^−^ = FeH_2_AsO_3_^+2^	7.282	2
H_3_AsO_3_ + 3 H_2_S = H_3_AsS_3_ + 3 H_2_O	7.75	3
H_3_AsS_3_ = H_2_AsS_3_^−^ + H^+^	−2.14	3
H_2_AsS_3_^−^ = HAsS_3_^−2^ + H^+^	−7.19	3
H_3_AsO_3_ + H_2_S = H_3_AsSO_2_ + H_2_O	4.68	3
H_3_AsSO_2_ = H_2_AsSO_2_^−^ + H^+^	−5.26	3
Aqueous As(V) species		
H_2_AsO_4_^−^ = H_2_AsO_3_^−^ + 0.5 O_2_	−30.53	1
H_2_AsO_4_^−^ + H^+^ = H_3_AsO_4_	2.29	1
H_2_AsO_4_^−^ = HAsO_4_^−2^ + H^+^	−6.99	1
H_2_AsO_4_^−^ = AsO_4_^−3^ + 2 H^+^	−18.79	1
Na^+^ + H_2_AsO_4_^−^ = NaH_2_AsO_4_	−1.775	2
K^+^ + H_2_AsO_4_^−^ = KH_2_AsO_4_	−1.895	2
Ca^+2^ + H_2_AsO_4_^−^ = CaH_2_AsO_4_^+^	1.495	2
Fe^+2^ + H_2_AsO_4_^−^ = FeH_2_AsO_4_^+^	2.795	2
Fe^+3^ + H_2_AsO_4_^−^ = FeH_2_AsO_4_^+2^	4.265	2
Na^+^ + H_2_AsO_4_^−^ = NaHAsO_4_^−^ + H^+^	−6.286	2
K^+^ + H_2_AsO_4_^−^ = KHAsO_4_^−^ + H^+^	−6.426	2
Ca^+2^ + H_2_AsO_4_^−^ = CaHAsO_4_ + H^+^	−4.466	2
Fe^+2^ + H_2_AsO_4_^−^ = FeHAsO_4_ + H^+^	−3.606	2
Fe^+3^ + H_2_AsO_4_^−^ = FeHAsO_4_^+^ + H^+^	2.975	2
Na^+^ + H_2_AsO_4_^−^ = NaAsO_4_^−2^ + 2 H^+^	−13.855	2
K^+^ + H_2_AsO_4_^−^ = KAsO_4_^−2^ + 2 H^+^	−13.995	2
Ca^+2^ + H_2_AsO_4_^−^ = CaAsO_4_^−^ + 2 H^+^	−12.62	2
Fe^+2^ + H_2_AsO_4_^−^ = FeAsO_4_^−^ + 2 H^+^	−11.151	2
Fe^+3^ + H_2_AsO_4_^−^ = FeAsO_4_ + 2 H^+^	−4.595	2
Arsenic solids		
AsS (realgar) + 2.5 H_2_O + 0.25 O_2_ = H_2_AsO_3_^−^ + 2 H^+^ + HS^−^	−7.78	1
As_2_S_3_ (orpiment) +6 H_2_O = 2 H_2_AsO_3_^−^ + 3 HS^−^ + 5 H^+^	−64.70	3
As_2_S_3_ (am) +6 H_2_O = 2 H_2_AsO_3_^−^ + 3 HS^−^ + 5 H^+^	−62.51	3
As_2_O_3_ (arsenolite) + 3 H_2_O = 2 H^+^ + 2 H_2_AsO_3_^−^	−19.72	1
As_2_O_3_ (claudetite) + 3 H_2_O = 2 H^+^ + 2 H_2_AsO_3_^−^	−19.75	1
As_2_O_5_ +3 H_2_O = + 2 H^+^ + 2 H_2_AsO_4_^−^	3.64	1
Ca_4_(OH)_2_(AsO4)_2_(H_2_O)_4_ + 6 H^+^ = 4 Ca^+2^ + 2 H_2_AsO_4_^−^ + 6 H_2_O	33.26	2
Ca_5_(OH)(AsO_4_)_3_ + 7 H^+^ = 5 Ca^+2^ + 3 H_2_AsO_4_^−^ + H_2_O	29.28	2
Ca_3_(AsO_4_)_2_(H_2_O)_2_ + 4 H^+^ = 3 Ca^+2^ + 2 H_2_AsO_4_^−^ + 2 H_2_O	13.37	2
Ca_3_(AsO_4_)_2_(H_2_O) + 4 H^+^ = 3 Ca^+2^ + 2 H_2_AsO_4_^−^ + H_2_O	15.96	2
CaHAsO_4_(H_2_O) + H^+^ = Ca^+2^ + H_2_AsO_4_^−^ + H_2_O	13.72	2
Fe_3_(AsO_4_)_2_ (symplesite) = 3 Fe^+2^ + 2 AsO_4_^−3^	−31.76	4
FeAsO_4_:2H_2_O (scorodite) = Fe^+3^ + AsO_4_^−3^ + 2 H_2_O	−25.89	5
FeAsO_4_:2H_2_O = Fe^+3^ + AsO_4_^−3^ + 2H_2_O	−23.16	5
CaAs_2_O_4_ + 2 H_2_O = Ca^+2^ + 2 H_2_AsO_3_^−^	−7.14	6
CaHAsO_3_:0.5H_2_O + H^+^ = Ca^+2^ + H_2_AsO_3_^−^ + 0.5 H_2_O	4.69	6

^a^Equilibrium constants at 25 °C, values recalculated for internal consistency

^b^(1) Nordstrom and Archer (2003), (2) Marini and Accornero (2007), (3) Vlassopoulos et al. (2010), (4) Johnston and Singer (2007), (5) Langmuir et al. (2006), (6) Nishimura and Robins (1998)

## Appendix 2

See Table 8.

**Table 8:** Thermodynamic equilibrium constants for mercury added to default LLNL database

Reaction	Log K^a^	Reference^b^	Comment
Mercury inorganic complexes			
Hg^+2^ + H_2_O = HgOH^+^ + H^+^	−3.4	1	
Hg^+2^ + 2 H_2_O = Hg(OH)_2_ + 2 H^+^	−5.98	1	
Hg^+2^ + 3 H_2_O = Hg(OH)_3_^−^ + 3 H^+^	−21.1	1	
Hg^+2^ + Cl^−^ = HgCl^+^	7.31	1	
Hg^+2^ + 2 Cl^−^ = HgCl_2_	14	1	
Hg^+2^ + 3 Cl^−^ = HgCl_3_^−^	14.93	1	
Hg^+2^ + 4 Cl^−^ = HgCl_4_^−2^	15.54	1	
Hg^+2^ + Cl^−^ + H_2_O = HgOHCl + H^+^	4.27	1	
Hg^+2^ + CO_3_^−2^ = HgCO_3_	11.46	1	
Hg^+2^ + OH^−^ + CO_3_^−2^ = Hg(OH)CO_3_^−^	19.32	1	
Hg^+2^ + CO_3_^−2^ + H^+^ = HgHCO_3_^+^	15.79	1	
Hg^+2^ + SO_4_^−2^ = HgSO_4_	2.4	1	
Mercury sulfide complexes			
(DOM)^−^ + H^+^ = (DOM)H	10	2	
2 (DOM)H + Hg^+2^ = Hg((DOM))_2_ + 2 H^+^	22	2	
H_2_O + H_2_S + Hg^+2^ = HOHgSH + 2 H^+^	19.4	2	c
2 H_2_S + Hg^+2^ = Hg(SH)_2_ + 2 H^+^	23.7	2	
2 H_2_S + Hg^+2^ = HgS_2_H^−^ + 3 H^+^	17.5	2	
2 H_2_S + Hg^+2^ = HgS_2_^−2^ + 4 H^+^	9.2	2	
Methylmercury and dimethylmercury			
(DOM)H + CH_3_Hg^+^ = CH_3_Hg((DOM)) + H^+^	6.5	2	
H_2_S + CH_3_Hg^+^ = CH_3_HgSH + H^+^	7.5	2	
CH_3_Hg^+^ + CH_4_ = CH_3_HgCH_3_ + H^+^	−10	3	
CH_3_Hg^+^ + Cl^−^ = CH_3_HgCl	5.25	3	
CH_3_Hg^+^ + H_2_O = CH_3_HgOH + H^+^	−4.63	3	
CH_3_Hg^+^ + CO_3_^−2^ = CH_3_HgCO_3_^−^	6.1	3	
CH_3_Hg^+^ + SO_4_^−2^ = CH_3_HgSO_4_^−^	0.94	3	
2 CH_3_Hg^+^ + H_2_O = CH_3_Hg_2_OH^+^ + H^+^	1.47	3	
Mercury solid phases			
HgS + 2 H^+^ = H_2_S + Hg^+2^	−29.4	2	d
Mercury exchange reactions			
H^+^ + (SOM)^−^ = H(SOM)	10	2	e
2 H(SOM) + Hg^+2^ = Hg((SOM))_2_ + 2 H^+^	19.5	2	e,f
H(SOM) + CH_3_Hg^+^ = CH_3_Hg((SOM)) + H^+^	6.5	2	e

^a^Equilibrium constants at 25 °C; values recalculated for internal consistency

^b^(1) Powell et al. (2005), (2) Skyllberg (2008), (3) Stumm and Morgan (1996)

^c^Experimental value for DOM-thiol sites from equation 10 (Skyllberg 2008)

^d^Metacinnabar

^e^Surface exchange constants for SOM thiol sites

^f^Skyllberg (2008) converted to conditional exchange constant using the Gaines–Thomas convention
